# Sleep, Cognitive Aging, and Dementia Risk: Insights From Longitudinal Cohort Studies

**DOI:** 10.1093/geroni/igaf122.1960

**Published:** 2025-12-31

**Authors:** Junxin Li, Jing Huang, Christopher Kaufmann

## Abstract

Healthy sleep is recognized as a key determinant of cognitive aging; however, its precise role remains unclear due to the heterogeneity of health conditions in older adults. This symposium brings together five studies leveraging large-scale, longitudinal cohort data to examine how sleep, independently or in combination with other conditions, influences cognitive aging and dementia risk. Additionally, it explores how cognitive performance profiles predict sleep and key health outcomes for independent living in older adults without dementia. The first presentation introduces a novel approach to measuring long-term sleep variability in adults aged 80 and older, providing insights into sleep patterns in the oldest-old population. The second study investigates how sleep disturbances and physical frailty jointly impact dementia risk in individuals with early cognitive concerns. The third study examines the combined effects of poor sleep and depression on dementia onset, emphasizing the need for multidimensional interventions. The fourth presentation explores sleep as a predictor of motoric cognitive risk in older adults, an early indicator of dementia. Finally, the last study identifies distinct cognitive profiles across six cognitive domains and evaluates their predictive value for key health outcomes essential for independent living, including sleep difficulties, depressive symptoms, falls, and daily functional impairment. Together, these studies highlight the critical role of sleep in cognitive aging and dementia prevention, identifying key risk factors and intervention targets across preclinical and early symptomatic stages of dementia.

